# Membrane association and release of wild-type and pathological tau from organotypic brain slice cultures

**DOI:** 10.1038/cddis.2017.97

**Published:** 2017-03-16

**Authors:** Cara L Croft, Matthew A Wade, Ksenia Kurbatskaya, Pavlina Mastrandreas, Martina M Hughes, Emma C Phillips, Amy M Pooler, Michael S Perkinton, Diane P Hanger, Wendy Noble

**Affiliations:** 1 Maurice Wohl Clinical Neuroscience Institute, Department of Basic and Clinical Neuroscience, Institute of Psychiatry, Psychology and Neuroscience, King's College London, London SE5 8AF, UK; 2 AstraZeneca Neuroscience iMED, Aaron Klug Building, Granta Park, Cambridge CB21 6GH, UK

## Abstract

The spatiotemporal transmission of pathological tau in the brain is characteristic of Alzheimer's disease. Release of both soluble and abnormal tau species from healthy neurons is increased upon stimulation of neuronal activity. It is not yet understood whether the mechanisms controlling soluble tau release from healthy neurons is the same as those involved in the spread of pathological tau species. To begin to understand these events, we have studied tau distribution and release using organotypic brain slice cultures. The slices were cultured from postnatal wild-type and 3xTg-AD mice for up to 1 month. Tau distribution in subcellular compartments was examined by western blotting, and tau release into culture medium was determined using a sensitive sandwich ELISA. We show here that 3xTg-AD cultures have an accelerated development of pathological tau abnormalities including the redistribution of tau to synaptic and membrane compartments. The 3xTg-AD slice cultures show elevated basal tau release relative to total tau when compared with wild-type cultures. However, tau release from 3xTg-AD slices cannot be further stimulated when neuronal activity is increased with potassium chloride. Moreover, we report that there is an increased pool of dephosphorylated membrane-associated tau in conditions where tau release is increased. These data suggest that there may be differential patterns of tau release when using integrated slice culture models of wild-type and transgenic mouse brain, although it will be important to determine the effect of tau overexpression for these findings. These results further increase our knowledge of the molecular mechanisms underlying tau release and propagation in neurodegenerative tauopathies.

The characteristic progressive accumulation of tau pathology in affected brain regions during the development of Alzheimer's disease (AD) is believed to result from the spread of aggregated tau along anatomically connected pathways.^1, 2, 3^ Restricted expression of transgenic tau in the entorhinal cortex results in both local accumulation of aggregated tau resulting in neuron loss, and the trans-synaptic spread of tau aggregates to more distal regions of the brain.^4, 5^ The spread of pathological tau and the extent of neurotoxicity in this model is also further accelerated by the presence of A*β*.^6^ The precise mechanisms of tau spread are not yet fully understood, however, the species of tau involved appears to be critical,^2^ as are the extracellular vesicles such as exosomes in which tau can be compartmentalised in different phosphorylation states.^7, 8, 9^ Synaptic connectivity, rather than the proximity of neurons, appears to be critical for tau propagation,^10^ and therefore understanding the routes by which tau is released from neurons is important for elucidating how pathological tau spreads through the brain in AD and other tauopathies.

We previously showed that tau release from neurons is a physiological process that is stimulated by neuronal activity in cultured rat neurons.^11^ Similar findings have since been reported *in vivo*.^12^ The relationship between tau release and neuronal activity appears to be bi-directional since both extracellular tau and A*β* feedback to neurons to perpetuate further tau release.^13^ Released endogenous tau is reported to be released free in a dephosphorylated full-length form^11^ or as N-terminally truncated fragments.^13, 14^ A small proportion of tau released under these conditions is associated with ectosomes, plasma membrane-derived vesicles.^15^ Particularly when tau is exogenously expressed or in a highly phosphorylated misfolded state, it is released from cells in association with exosomes^7, 16^ and can be highly phosphorylated.^17^ The apparent discrepancies in these findings raise the question of whether physiological species of soluble tau are released via the same mechanisms as are involved in the propagation of pathological tau forms.

To investigate these mechanisms further, we have examined tau release from organotypic brain slice cultures prepared from wild-type and 3xTg-AD mice.^18, 19^ Recent publications have highlighted the utility of using organotypic nervous tissue slices for modelling neurodegenerative diseases. Organotypic brain slice cultures from transgenic models of AD develop some of the main molecular hallmarks of human disease including 'plaque-like' depositions when prepared from APP transgenic mice,^20^ and abnormally phosphorylated tau when tau overexpressing mice are used.^21, 22^ Moreover, these models can be used to examine the cellular mechanisms underlying AD including A*β*-mediated synaptotoxicity.^23, 24, 25, 26^

We show here for the first time that organotypic brain slices cultured from postnatal 3xTg-AD pups show accelerated development of AD-like molecular pathology. Notably, 3xTg-AD slices exhibit increased basal tau release relative to total tau amounts when compared with wild-type slices. Stimulation of neuronal activity with KCl increased tau release from wild-type slices, in line with reports in primary neurons and *in vivo*,^11, 12^ whereas the extent of tau release from 3xTg-AD slices was unchanged by KCl stimulation. In both wild-type and 3xTg-AD slices, there was a marked correlation between the amount of dephosphorylated tau present at membranes and the extent of tau release, in keeping with reports that tau localisation is important for its propagation.^27^ Taken together, these results suggest that the mechanisms governing the release of physiological and pathological forms of tau may be differentially regulated by neuronal stimulation. These findings might support the existence of functionally distinct pools of extracellular tau with physiological tau contributing to neuronal signalling in a healthy brain environment^28^ and pathological tau driving the spread of tau pathology in diseased brain. However, potential effects of overexpression on tau localisation and release must be taken into account when interpreting the results of this work as it is possible that saturation of synaptic activity is responsible for the lack of effect of neuronal stimulation on tau release from 3xTg-AD slices.

## Results

### Organotypic brain slice cultures from 3xTg-AD mice recapitulate molecular phenotypes of aged brain

**Figure 1:**
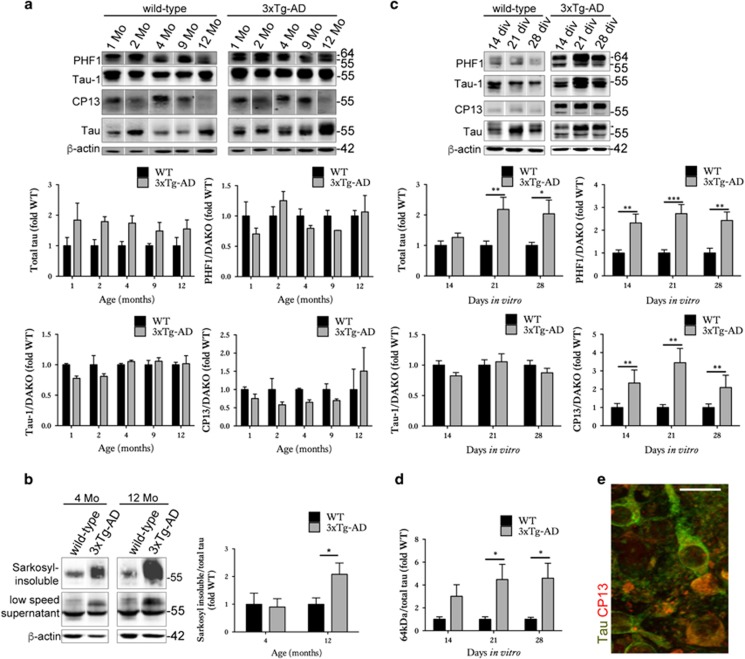
Organotypic brain slice cultures from 3xTg-AD mice show accelerated development of tau abnormalities. Representative western blots of cortical brain lysates prepared from 1-, 2-, 4-, 9- and 12-month-old wild-type and 3xTg-AD mice, probed with antibodies against tau (both non-phosphorylated and phosphorylated), PHF1 (phospho-Ser396/404), Tau-1 (dephospho-Ser199/202/Thr205) and CP13 (phospho-Ser202). Blots were also probed with an antibody against *β*-actin as a loading control. Bar charts show amounts of total tau relative to *β*-actin, and phospho-tau as a proportion of total tau, *n*=3. (**b**) Sarkosyl-insoluble tau was isolated from 4- and 12-month-old wild-type (WT) and 3xTg-AD mice. Western blots show tau in the low-speed supernatant and sarkosyl-insoluble fraction. The amount of sarkosyl-insoluble relative to total tau in low-speed supernatants is shown in the bar chart, *n*=3. (**c**) Lysates were prepared from wild-type and 3xTg-AD brain slice cultures at 14, 21 and 28 days *in vitro* (DIV), *n*=12. Blots were probed with total tau and phosphorylation-dependent tau antibodies. An antibody against *β*-actin was used as a loading control. Bar charts show amounts of total tau relative to *β*-actin, and phospho-tau as a proportion of total tau (Dako), *n*=12. (**d**) Bar chart shows the amount of 64 kDa tau in wild-type and 3xTg-AD brain slice cultures as detected by the total tau antibody (indicated by arrowhead), quantified as a proportion of total tau, *n*=12. (**e**) Immunohistochemistry showing neurons in 28 DIV 3xTg-AD slices labelled with antibodies against total tau and tau phosphorylated at Ser202 (CP13). Scale bar is 20 *μ*m. Data for all graphs are mean±S.E.M. and is shown as fold change from wild-type(fold WT). *P<0.05, **P<0.01, ***P<0.001

**Figure 2:**
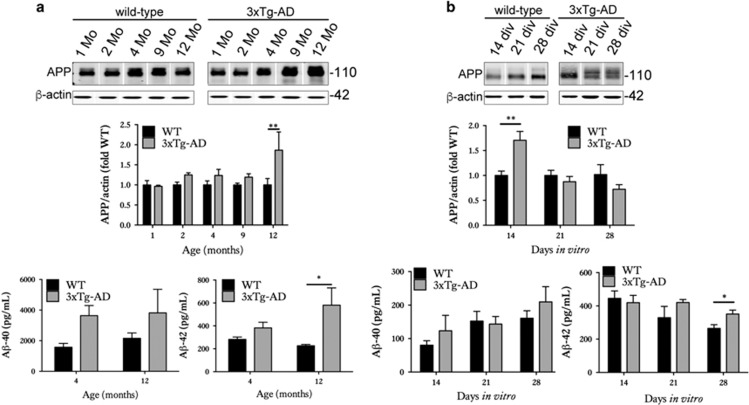
Organotypic brain slice cultures from 3xTg-AD mice show altered APP and A*β*. (**a**) Representative western blots of cortical brain lysates prepared from 1-, 2-, 4-, 9- and 12-month-old wild-type and 3xTg-AD mice, probed with an antibody against N-terminal APP (22c11). Blots were also probed with an antibody against *β*-actin as a loading control. Bar chart shows amounts of APP relative to *β*-actin as fold change from wild-type (fold WT). A*β* ELISAs were used to measure A*β*1-40 and A*β*1-42 content. Bar charts show A*β* amounts as pg/ml, *n*=3. (**b**) Lysates were prepared from wild-type and 3xTg-AD brain slice cultures at 14, 21 and 28 days *in vitro* (DIV), *n*=12. Blots were probed with the 22c11 antibody against APP. An antibody against *β*-actin was used as a loading control. Bar chart shows amounts of APP relative to *β*-actin in each sample as fold change from control (fold WT), *n*=9. A*β*1-40 and A*β*1-42 amounts as pg/ml in brain slice cultures are also shown, *n*=12. Data are mean±S.E.M. *P<0.05, **P<0.01

We first determined the extent to which organotypic brain slice cultures prepared from 3xTg-AD mice recapitulate the neurodegenerative molecular changes that develop *in vivo*^18, 19^ and in human AD brain.^29^ Cortical extracts from 3xTg-AD mice showed overexpression of total tau, as expected on the basis of human tau overexpression (Figure 1a). At the ages examined here, no significant increases in tau phosphorylation at CP13 (Ser202) or PHF1 (Ser396/404) were observed, in line with previous reports.^30^ However, an increased abundance of sarkosyl-insoluble tau was apparent in 12-month-old 3xTg-AD cortex (Figure 1b), as described previously.^18, 19, 31, 32^ The 3xTg-AD cortex also showed a progressive accumulation of total APP and A*β*1-42 relative to age-matched wild-type controls (Figure 2a), similar to that previously described by Oddo *et al.*^19^

Cultured brain slices from 3xTg-AD mice were characterised by an accumulation of total tau (Figure 1c), similar to that observed in aged brain.^19^ 14–28 days *in vitro* (DIV) slices also displayed significant increases in tau phosphorylation at Ser202 and Ser396/404 relative to wild-type controls, with these epitopes being hyperphosphorylated in AD brain.^33^ These data suggest accelerated disease-associated tau phosphorylation in cultured brain slices in comparison to *in vivo* aged 3xTg-AD brain, as previously reported for other tau transgenic lines.^22^ In support of this, an accumulation of approximately 64 kDa tau (Figure 1c) was apparent in 3xTg-AD slices at 21 DIV (Figure 1d). Such higher molecular weight tau species precede the accumulation of sarkosyl-insoluble material in several tau transgenic lines.^34, 35^ Tau in 3xTg-AD slices was not limited to axons but showed a somatodendritic appearance, characteristic of tau localisation in AD (Figure 1e). 3xTg-AD brain slice cultures also showed increased expression of APP at 14 DIV and over-production of A*β*1-42 when aged for 28 DIV (Figure 2b). It is possible that the increased APP observed in slice cultures at relatively early DIV represents a synaptic compensation response, similar to that recently reported during AD progression.^29^ We have previously shown that sustained culture over months allows the development of small diffuse plaques in other APP transgenic lines.^22^

**Figure 3:**
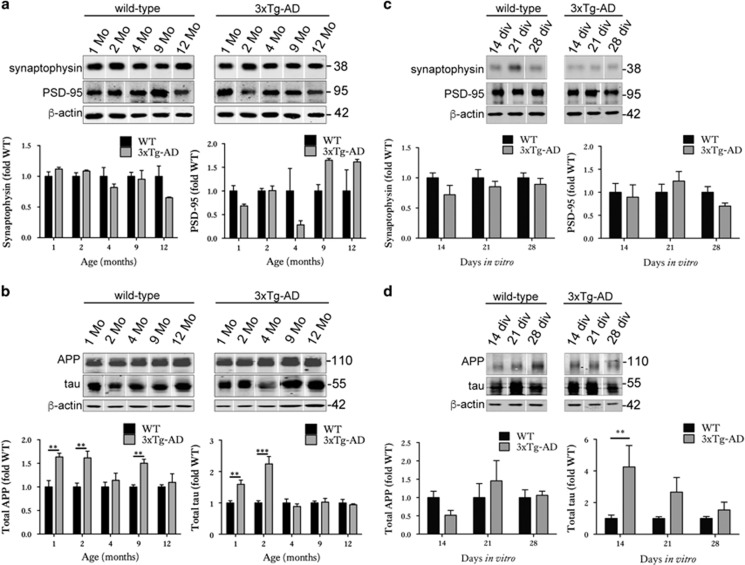
Organotypic brain slice cultures from 3xTg-AD mice accumulate tau in synaptic compartments. Representative western blots of proteins in the synaptosome fractions prepared from 1-, 2-, 4-, 9- and 12-month-old wild-type and 3xTg-AD brain. Blots were probed with antibodies against (**a**) synaptophysin and PSD-95 and (**b**) APP (22c11) and total tau, *n*=3. Bar charts show amounts of protein in the synaptic fraction following normalisation to *β*-actin in each sample. Western blots of proteins in the synaptosome fractions prepared from wild-type (WT) and 3xTg-AD brain slice cultures at 14, 21 and 28 DIV. Blots were probed with antibodies against (**c**) synaptophysin and PSD-95 and (**d**) APP (22c11) and total tau, *n*=9. An antibody against *β*-actin was used as a loading control. Bar charts show amounts of protein in the synaptic fraction. Data are shown as fold change from wild-type at each time point. Data are mean±S.E.M. **P<0.01, ***P<0.001

Synaptosomes were prepared from mouse brain and slice cultures to allow examination of protein association with synaptic compartments. We have previously demonstrated the enrichment of synaptic proteins in the synaptosomal fraction relative to the non-synaptosomal fraction using this protocol.^36^ No changes in post-synaptic markers, even in the oldest 3xTg-AD animals examined here (12 months of age, Figure 3a), were detected in synaptosomes isolated from 3xTg-AD and wild-type cortex. This is in keeping with previous analysis reporting no alterations in synapse density or synapse contact area in 13-month-old 3xTg-AD mice.^37^ Interestingly, when synaptosome preparations were immunoblotted with antibodies against tau and APP, the results suggested a transient trend towards upregulation of tau in the synaptic compartment in young (1–2-month-old) mice that was lost with aging (Figure 3b). APP was also somewhat increased in synapses; the increases at 1, 2 and 9 months being significantly different when compared with wild-type (Figure 3b).

In 3xTg-AD slices, no reductions in PSD-95 and synaptophysin amounts were apparent in immunoblotted synaptosomes, suggesting that synapses remaining in these cultures are relatively healthy and intact (Figure 3c). However, these slice cultures also showed a transient redistribution of tau into synaptic compartments in 14 DIV slices relative to controls (Figure 3d). This likely represents a redistribution rather than passive process as tau levels in the synapses are not sustained, unlike the levels of tau overexpression observed in 3xTg-AD slices that are only significantly increased from wild type from 21 DIV (Figure 1c). Such redistribution of tau to synapses was recently shown to be an important biological correlate of dementia in AD,^38^ and suggests that some AD-relevant changes may be recapitulated in this slice culture model. There were no changes in APP abundance at synapses over 28 days in culture (Figure 3d). Again, the abundance of APP at synapses does not appear to be associated with APP overexpression in these slices since total APP was significantly elevated relative to wild type at 14 DIV, at which time there was no increase in synaptic APP.

### 3xTg-AD slice cultures show increased levels of basal tau release

We next assessed basal tau release from 3xTg-AD slice cultures in comparison with that from wild-type controls. Total amounts of tau released into medium over a period of 30 min were measured using an in-house total tau ELISA that uses two commercially available tau antibodies with epitopes in the middle region (BT2) and C-terminal half (Dako) of tau. Intracellular tau amounts were determined in the same cultures by immunoblotting, as described above. The amounts of tau detected in culture medium were then normalised to amounts of intracellular tau in the same sample to control for the effects of tau overexpression in the 3xTg-AD slices and to allow the proportion of total tau released from slices to be determined. In addition, the amount of lactate dehydrogenase (LDH) released into the media from the same slices was measured to ensure that changes in tau release are not a result of increased cell death. The LDH content in culture medium was calculated as a proportion of the total LDH in the slice culture lysates plus that measured in medium.

**Figure 4:**
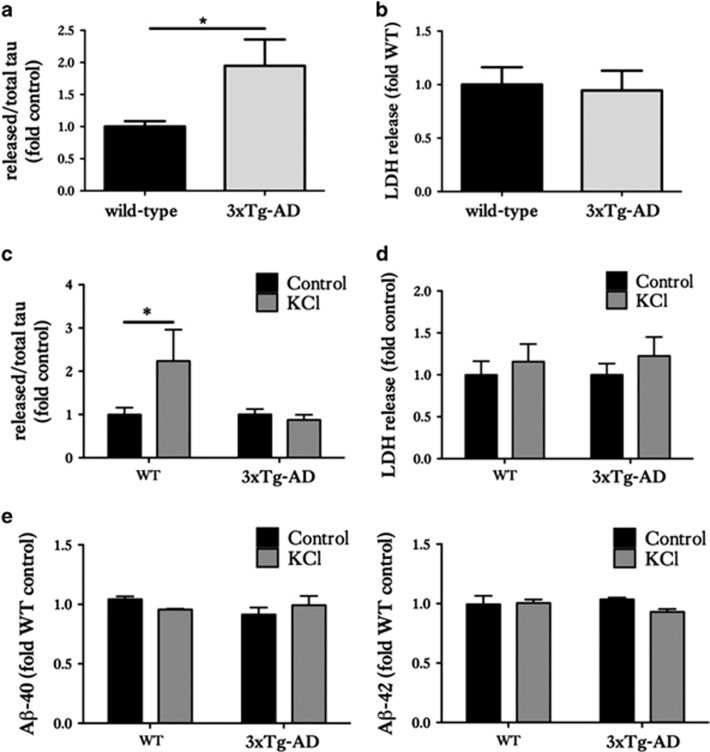
Organotypic brain slice cultures from 3xTg-AD and wild-type mice show differential tau release responses to neuronal stimulation. Tau and LDH release into the culture medium of 28 DIV wild-type (WT) and 3xTg-AD slice cultures were measured. (**a**) Bar chart shows amounts of tau released into the media under basal (non-stimulated) conditions, after standardisation to total intracellular tau amounts in the same sample. (**b**) Release of lactate dehydrogenase (LDH) into media under basal conditions was measured as an indicator of slice viability. (**c**) Tau release into culture medium following stimulation with control or 50 mM KCl for 30 mins and (**d**) LDH release under these conditions. (**e**) Bar charts show A*β*1-40 and A*β*1-42 amounts in culture medium following treatment with 50 mM KCl or control. Data are shown as fold change from WT slice cultures in (**a** and **b**). Data are fold change from control treatment in (**c** and **d**). Mean±S.E.M. is shown, *n*=12–24, *P<0.05

The 3xTg-AD slice cultures were found to release a significantly larger proportion of tau under basal conditions compared with wild-types (Figure 4a). There were no significant differences in LDH release (Figure 4b). These data suggest that disease-associated forms of tau are released from healthy neurons more readily than ‘normal' tau under basal conditions.

### Neuronal stimulation increases tau release from wild-type, but not 3xTg-AD brain slice cultures

To assess the effects of neuronal stimulation on the release of normal and disease-associated tau from organotypic brain slice cultures, 50 mM KCl was applied to depolarise neurons and stimulate neuronal activity over a period of 30 min. No significant differences in LDH release between depolarised and non-depolarised wild-type and 3xTg-AD slice cultures was found, suggesting any increased tau release was not a result of increased cell death (Figure 4d). When stimulated with KCl, wild-type slice cultures showed significant increases in tau release compared with non-stimulated wild-type slice cultures (Figures 4c, *P*<0.05), in agreement with published data from dissociated rodent cells^11^ and *in vivo* in wild-type mice.^12^ In contrast, 3xTg-AD slice cultures stimulated with KCl did not demonstrate any further release of tau when compared with non-stimulated 3xTg-AD slice cultures (Figure 4d).

Bright *et al.*^13^ reported that secreted human tau positively feeds back to neurons to cause further neuronal activity and stimulate A*β* production over the course of 20 days. We therefore measured A*β* amounts in slice cultures stimulated with KCl for 30 min, but we found no changes in the amount of A*β*1-40 or A*β*1-42 in KCl-treated cultures in comparison with controls (Figure 4e). It is possible that longer periods of stimulation may have affected A*β* release from slices, but this was not determined here.

### The presence of dephosphorylated tau at neuronal membranes is associated with the extent of tau release from brain slice cultures

**Figure 5:**
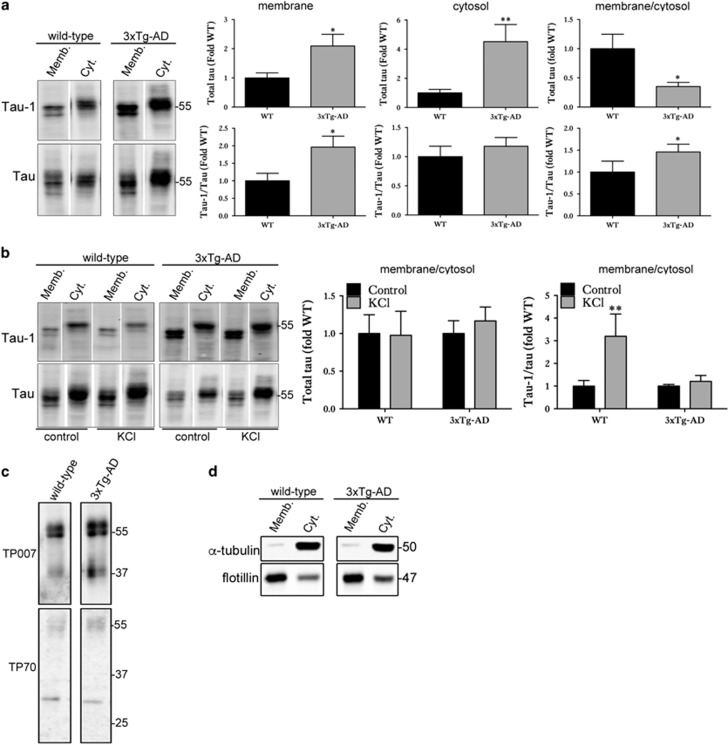
Association of dephosphorylated tau at membranes with tau release. (**a**) Representative western blots of membrane and cytosol fractions prepared from WT and 3xTg-AD slice cultures under basal conditions showing total (phosphorylated and non-phosphorylated) tau and tau dephosphorylated at Ser199/202/Thr205 (Tau-1). Bar charts show relative total tau amounts in the membrane and cytosolic fraction and the ratio of membrane/cytosolic tau. The amount of Tau-1 as a proportion of total tau is also shown for these fractions. Data are shown as fold change from wild-type (WT) slice cultures, *n*=12. (**b**) Representative western blots of membrane and cytosol fractions prepared from WT and 3xTg-AD slice cultures after stimulation with 50 mM KCl or vehicle for 30 min. Blots showing amounts of total tau and tau dephosphorylated at the Tau-1 epitope relative to total tau (Dako) in membrane and cytosolic fractions are shown, as well as graphs showing the ratio of membrane/cytosol tau. Data are shown as fold change from wild-type (WT). Data are mean±S.E.M., *n*=12, *P<0.05, **P<0.01. (**c**) Representative western blots of membrane fractions prepared from 28 DIV wild-type and 3xTg-AD slice cultures showing N-terminally intact (TP007) and C-terminally intact (TP70) tau at 50–64 kDa. Some smaller fragments of tau were apparent at approximately 37 and 30 kDa. (**d**) Western blots showing enrichment of the membrane protein flotillin and the cytosolic protein *α*-tubulin in the membrane and cytosolic fractions, respectively

Although generally considered to be cytosolic, a significant proportion of tau is associated with plasma membranes.^39, 40^ Membrane-associated tau is predominantly dephosphorylated at serine and threonine residues.^40, 41^ As some of the vesicles (ectosomes, exosomes) associated with tau release originate at the plasma membrane, it was of interest to investigate the proportions of tau at membranes in wild-type and 3xTg-AD slices to determine whether there is any association with tau release. Differential centrifugation was used to prepare membrane and cytosol fractions that were then immunoblotted with antibodies against tau. In 3xTg-AD slice cultures, both the membrane and cytosolic fractions contained significantly more tau than controls due to tau overexpression in this line. However, when the ratio of membrane:cytosolic tau was calculated, 3xTg-AD slices had a significantly smaller proportion of tau associated with membranes when compared with that in wild-type slice cultures (Figure 5a). However, the proportion of tau dephosphorylated at the Tau-1 epitope was increased in the membrane fraction of 3xTg-AD slices compared with controls (Figure 5a), an environment in which these slices also show elevated tau release relative to that of wild-type slice cultures.

Membrane and cytosolic fractions were next prepared from wild-type and 3xTg-AD slice cultures at 28 DIV treated with vehicle or 50 mM KCl to induce depolarisation. In both wild-type and 3xTg-AD slice cultures treated with KCl, the ratio of total tau present in the membrane fraction relative to the cytosolic fraction did not differ from control treatment (Figure 5b). However, in wild-type slice cultures treated with KCl, the amount of membrane-associated tau dephosphorylated at the Tau-1 epitope was significantly increased compared with control treatment (Figure 5b), indicating an increased association of dephosphorylated tau with membranes following neuronal stimulation, conditions under which tau release was increased.

In contrast, in 3xTg-AD slice cultures treated with KCl, no changes in membrane-associated tau dephosphorylated at Tau-1 were observed (Figure 5b), conditions under which tau release is also not increased above basal levels. Taken together, these data suggest that conditions leading to an increased pool of dephosphorylated tau at membranes are associated with elevated tau release from slice cultures. In addition, these data further suggest that differences in the effect of neuronal stimulation on tau release from tissues containing normal or disease-associated tau might be related to the subcellular localisation of pools of dephosphorylated tau.

The cleavage of tau is a post-translational modification, which can affect tau function, with some fragments acting as seeds which promote tau aggregation,^42^ altered tau clearance, and, *in vivo*, changes in cognition and motor ability.^43^ Tau cleaved at both its N- and C-termini has been detected in AD brain.^44^ Currently, no consensus exists on whether intact, N-terminally or C-terminally truncated tau is the species of tau that propagates. Endogenous tau released under basal conditions from primary cortical rat neurons is predominantly full length,^11^ although some truncated species have been identified together with full-length tau.^15^ However, others show that only C-terminally truncated forms of endogenous tau are secreted from unstimulated human and rodent neurons.^14^ In contrast, pathological extracellular tau in a rat model of tauopathy contains tau lacking the C terminus but containing the N terminus,^15^ and C-terminally truncated tau is released from AD synaptosomes.^45^ In addition, exogenously expressed hyperphosphorylated tau secreted from non-neuronal cells was demonstrated to be C-terminally cleaved.^17^

To determine which species of tau are associated with membranes, the membrane fractions prepared from 28 DIV wild-type and 3xTg-AD slice cultures were immunoblotted with primary antibodies directed against the N terminus (TP007) and C terminus (TP70) of tau.^46, 47^ Membranes of both wild-type and 3xTg-AD slice cultures contain predominantly full-length tau species of 50–64 kDa with intact N- and C- termini (Figure 5c). However, some smaller tau species of approximately 35–40 kDa and 28–30 kDa were detected by the TP007 and TP70 antibodies, respectively, indicating that some C-terminally and N-terminally truncated tau species are also present at membranes in both wild-type and 3xTg-AD slices. There were no marked differences in the tau species detected between genotypes, indicating that phosphorylation or conformation of tau is likely more important for determining the dynamics of tau release from these slice cultures. Finally, to confirm the stringency of the fractionation, cytosolic and membrane preparations were probed for membrane-associated flotillin and cytosolic *α*-tubulin (Figure 5d). As expected, flotillin-1 was primarily localised in the membrane fraction, whereas *α*-tubulin was enriched in the cytosol.

## Discussion

The results presented show for the first time that slice cultures from 3xTg-AD mice show progressive and accelerated development of AD-like alterations in APP, tau and synaptic proteins that mirror pathology development in 3xTg-AD mice *in vivo* and human AD. In particular, we show the presence of highly phosphorylated and higher molecular weight tau species in 3xTg-AD slices relative to age-matched wild-type controls. Indeed, higher phosphorylation of tau was found in slices compared with *in vivo*. This appears to be a common phenomenon in primary and slice culture models that is likely to result from the stress of culture preparation and/or the presence of medium components that can affect tau phosphorylation signalling pathways, including insulin, glutamate and horse serum.^22^

Using this culture system as a means to probe the effect of pathological tau alterations on its pattern of release, we show that 3xTg-AD slices release almost two-fold the amount of tau released by wild-type slice cultures in basal conditions, after normalising against total amounts expressed in 3xTg-AD slices. It should be noted that it was not possible to account for possible differences in tau localisation or association with membrane-associated vesicles in these experiments. Overexpression of tau has previously been shown to result in release of highly phosphorylated tau^17^ from cells, often in association with exosomes^7, 16^ and it will be of great interest to next determine whether tau released from 3xTg-AD slices is aberrantly phosphorylated and/or cleaved relative to tau released from wild-type slices. Regardless of the species involved, it is likely that the extracellular tau, released by an as-yet undefined mechanism, contributes to tau propagation in disease as demonstrated previously in several AD and tauopathy models.^5, 48, 49, 50^

The results also demonstrate that 3xTg-AD slice cultures contain a greater proportion of dephosphorylated tau at membranes as shown by increased abundance of tau dephosphorylated at the Tau-1 site in this fraction. Although it has not been possible to explore the exact relationship between membrane-associated tau and tau release in this work, previous studies have suggested that tau is held at the membrane before it is released, potentially in association with exosomes^7, 8, 16^ or ectosomes.^15^ In agreement with our work, tau species at the membrane have previously been identified as being predominantly dephosphorylated at serine and threonine residues when compared with cytosolic tau,^40^ and increased phosphorylation of tau, particularly at N-terminal residues, reduces its association with the membrane.^41^ Taken together, this suggests that these largely dephosphorylated species of tau may be awaiting secretion, as it has been demonstrated that both physiological and pathological extracellular tau species are predominantly dephosphorylated compared with species found intracellularly.^11, 17^ It remains to be determined which species of tau are released by wild-type and 3xTg-AD slice cultures, and the precise mechanisms by which they are released.

Stimulating neuronal activity with KCl was shown here to significantly increase tau release from wild-type slice cultures, but not to stimulate any further increases in tau release from 3xTg-AD slice cultures. Increased release of tau after neuronal depolarisation has previously been shown in both wild-type rat primary cortical neurons^11^ and *in vivo* in wild-type mice.^12^ The reasons why KCl does not stimulate further tau release from 3xTg-AD slices are not yet identified, but may be a result of saturation of synaptic activity in 3xTg-AD slices as A*β* is known to cause increased cellular hyperexcitability.^47, 48^ Similarly, A*β* has been shown to accelerate the propagation of tau *in vivo*.^49^ Therefore, it is conceivable that increased synaptic activity in the 3xTg-AD slices, reflective of conditions in AD brain, underlies the differences in tau release observed here following neuronal stimulation. However, increased tau release was found to occur upon KCl depolarisation of synaptosomes isolated from human AD, but not control, brain,^45^ in apparent disagreement with the data presented here. In this context, it is worth remembering that 3xTg-AD mice express P301L FTLD-tau and tau-associated neurodegeneration appears to differ in AD and FTLD-tau. Synapse and cell loss is more abundant in FTLD despite the number of NFTs being markedly less than those found in affected regions of AD. Thus, it is important to replicate these findings using a mouse model that overexpresses wild-type human tau such as the htau^51^ or Tau35^43^ lines.

It should also be noted that Sokolow *et al.*^45^ measured tau release only from synapses, as compared with the integrated and functionally connected neural cells present in slice cultures. It is possible that the increased phosphorylation of tau in 3xTg-AD slices changes its subcellular distribution such that an increased pool of tau is docked at membranes in readiness for release, and this cannot be further increased by neuronal stimulation. In partial support of this idea, increased tau release upon KCl treatment of wild-type slices was associated with increased amounts of membrane-associated dephosphorylated tau. This might therefore argue that both physiological and pathological tau release occur, at least in part, via a common mechanism that involves accumulation of dephosphorylated tau at membranes. There were no changes in the amounts of dephosphorylated tau at membranes on KCl stimulation of 3xTg-AD slices, conditions under which no elevated tau release was observed.

The data presented here demonstrate that organotypic brain slice cultures from 3xTg-AD mice rapidly develop AD-relevant disease features and can be used as a model *in vitro* system to study some aspects of both tau and A*β* pathology. In addition, we demonstrate that slice cultures are an ideal model with which to investigate the processes associated with the propagation of tau in AD.

## Materials and methods

All materials were obtained from Sigma (Poole, Dorset, UK) unless otherwise stated.

### Organotypic brain slice culture

Organotypic brain slice cultures were prepared from postnatal day 8–9 3xTg-AD^18, 19^ and background control wild-type mice as previously described.^22^ Culture medium was changed every 2–3 days. The slices were treated after 28 days in culture.

### Cell death assays

Cytotoxicity was evaluated by measuring lactate dehydrogenase (LDH) in culture medium using Cytotox 96 assay kits (Promega, Madison, WI, USA) according to the manufacturer's directions. LDH content in medium and lysed slice cultures was measured to determine the total LDH content. Optical density was measured at 492 nm (Wallac 1420 Victor^3^ plate reader, PerkinElmer, Waltham, MA, USA). LDH release from slice cultures was calculated as a percentage of total LDH in each well.

### Preparation of slice culture lysates for western blotting

After treatments, the medium was removed and the slices were washed in ice-cold phosphate-buffered saline (PBS) followed by lysis in extra strong lysis buffer (10 mM Tris-HCl (pH 7.5), 0.5% (w/v) sodium dodecyl sulphate (SDS), 20 mM sodium deoxycholate, 1% (v/v) Triton X-100, 75 mM sodium chloride, 10 mM ethylenediaminetetraacetic acid (EDTA), 2 mM sodium orthovanadate, 1.25 mM sodium fluoride and protease inhibitor cocktail for mammalian tissues (Roche Diagnostics, Burgess Hill, UK)), and centrifugation at 16 000 × *g*_av_ for 20 min at 4 °C. The protein concentration of supernatants was measured using a BCA protein assay kit (Pierce Endogen, Rockford, USA) and the samples were standardised to equal protein concentration before being analysed by SDS-PAGE.

### Subcellular fractionation

Cytosol and membrane fractions were prepared as previously described.^40^ In brief, the cells were scraped into hypotonic buffer (10 mM sodium bicarbonate containing 20 mg/ml deoxyribonuclease I, 1 mM sodium orthovanadate and Complete protease inhibitor cocktail (Roche, Mannheim, Germany)) and disrupted by sonication (five strokes with probe sonicator) on ice. The lysates were centrifuged at 720 × *g*(av) for 5 min at 4 °C, to remove unbroken cells, and the resultant supernatants were centrifuged at 100 000 × *g*(av) for 1 h at 4 °C. The final supernatant (‘cytosolic fraction') was mixed with Laemmli sample buffer and the pellet (‘membrane fraction') was resuspended in the same buffer. The samples were normalised for total protein content following measurement of protein amounts using a BCA protein assay kit (Pierce Endogen, Rockford, IL, USA) before use.

### Preparation of synaptosomes

Synaptosomes were prepared as previously described.^52^ In brief, the slices were homogenised in synaptosome lysis buffer (10 mM Tris-HCl pH 7.4, containing 0.32 M sucrose, 2 mM EGTA, 2 mM EDTA and Complete protease inhibitor cocktail (Roche, Mannheim, Germany)) and centrifuged at 1000 × *g*(av) for 10 min at 4 °C to remove cell nuclei and debris. The supernatant (‘total fraction') was then centrifuged at 10 000 × *g*_av_ for 20 min at 4 °C. The final supernatant (‘non-synaptosomal') was mixed with Laemmli sample buffer and the pellet (‘synaptosomal fraction') was resuspended in the same buffer.

### Tau ELISA

The slice culture medium was replaced with Hank's Balanced Salt Solution (Life Technologies Ltd, Paisley, UK). The slices were treated with the indicated compounds diluted in HBSS. HBSS was collected from neurons and centrifuged at 12 000 g for 10 min at 4 °C to remove cell debris. Tau content in HBSS was determined by ELISA using a combination of a polyclonal total tau primary antibody (Dako, Ely, UK; cat #A0024 RRID:AB_10013724) and mouse monoclonal total tau antibody (BT2, Fisher Scientific, Loughborough, UK; cat #MN1010 RRID:AB_10975238) according to methods adapted from ref. 14. In brief, 96-well Nunc Maxi-Sorp plates were coated with 2 *μ*g/ml BT2 for 8 days at 4 **°**C with shaking. Starting blocking buffer (Thermo Scientific Ltd, Loughborough, UK) was added for 4 h at ambient temperature with shaking, followed by application of conditioned HBSS from slice cultures overnight at 37 **°**C. Following washing, the capture antibody (Dako) was added and incubated overnight at ambient temperature with shaking. Secondary antibody (horseradish peroxidase (HRP)-labelled anti-rabbit IgG (GE Healthcare, Little Chalfont, UK)) was added for 1 h at ambient temperature with shaking and a stabilised chromogen TMB substrate solution (Thermo Scientific Ltd, Loughborough, UK) was added for detection. Absorbance was read at 450 nm with a Wallac 1420 Victor^3^ multilabel plate reader (PerkinElmer).

### A*β* ELISA

Quantification of A*β*1-40 and A*β*1-42 in slice lysates was performed using ELISA kits from Invitrogen (Paisley, UK) (A*β*1-40 ELISA KHB3481; A*β*1-42 ELISA KHB3442) as we have previously described.^53^

### SDS-PAGE and immunoblotting

A total 5–20 *μ*g protein was separated on 10% or 12% (w/v) SDS-PAGE gels and electrophoretically transferred to nitrocellulose membrane, as we have described previously.^29^ White lines separating lanes in some immunoblots indicate removal of portions of the blots for clarity and/or splicing together of distinct blots. The following primary antibodies were used for western blotting. Total tau (rabbit IgG; Dako Ltd.; cat #A0024 RRID:AB_10013724); APP (mouse IgG1, clone 22c11; Millipore UK Ltd; Watford, UK; cat #AB5352 RRID:AB_91793); APP (mouse IgG1, clone 6E10; Covance Research Products; cat #SIG-39300-200 RRID:AB_10175290); synaptophysin (mouse IgM, clone SP15; Enzo Life Sciences, Exeter, UK; cat #ADI-VAM-SV011-F RRID:AB_11177905); PSD-95 (rabbit IgG, Cell signaling, Danvers, MA, USA; cat #2507 RRID:AB_561221); *β*-actin (mouse IgG1, clone AC-15, Abcam, Cambridge, UK; cat #ab40864 RRID:AB_722536). The following tau antibodies were kindly gifted by Peter Davies (Albert Einstein College of Medicine, Bronx, NY, USA): CP13 (phospho-Ser-202; mouse IgG1); PHF1 (phospho-Ser-396/404; mouse IgG1).^54^

### Immunohistochemistry

Organotypic brain slice cultures were fixed on their membrane inserts in 4% PFA for 4 h and stained according to Gogolla *et al.*^55^ In brief, slice cultures were cut while still on their membranes and then treated as free-floating sections. The slice cultures were permeabilised for 18 h in 0.5% Triton X-100 at 4 °C and then blocked in 20% bovine serum albumin (BSA) for 4 h at RT. The slice cultures were incubated in total tau and CP13 (as above) primary antibodies overnight at 4 °C in 5% BSA, washed and then incubated in fluorophore-coupled secondary antibodies for 4 h at RT. The slice cultures were washed a final time before mounting on slides with fluorescent mounting medium (Dako Ltd.). The slice cultures were imaged using an Eclipse Ti-E Inverted (Nikon Instruments, Kingston Upon Thames, UK) microscope using a CSU-X1 Spinning Disk Confocal and Andor Ixon3 EM-CCD camera imaging system setup using a 60 Plan Apo VC N2 objective lens (Nikon Instruments).

### Statistics

For slice culture and mouse brain characterisation, data were analysed by two-way ANOVA followed by Sidak's *post hoc* analysis. Tau release from wild-type and 3xTg-AD slice cultures was analysed by unpaired *t*-test. Tau release from these cultures following treatment with KCl and vehicle was assessed using two-way ANOVA followed by Bonferonni *post hoc* analysis. Differences were considered statistically significant when *P*<0.05. For slice cultures, *n* refers to each well which contained three slices.
